# Detection of *Ureaplasma parvum* in amniotic fluids via reanalysis of prenatal copy number variation sequencing data: an exploratory study

**DOI:** 10.3389/fcimb.2025.1579049

**Published:** 2025-08-14

**Authors:** Guan Wang, Weifen Chen, Xiaodan Chen, Hongying Hou, Jun Zhang, Zhenyan Han

**Affiliations:** ^1^ Department of Laboratory Medicine, Third Affiliated Hospital of Sun Yat-Sen University, Guangzhou, Guangdong, China; ^2^ Department of Obstetrics and Gynecology, Third Affiliated Hospital of Sun Yat-Sen University, Guangzhou, Guangdong, China; ^3^ Department of Bioinformatics, Guangzhou Forevergen Medical Laboratory Co., Ltd, Guangzhou, Guangdong, China

**Keywords:** intrauterine infection, *Ureaplasma parvum*, copy number variation sequencing, amniocentesis, pregnancy outcomes

## Abstract

**Background:**

Detecting microbes in amniotic fluids via amniocentesis represents the standard method for diagnosing intrauterine infections. Given its similarity to metagenomic next-generation sequencing, copy number variation sequencing (CNV-seq) data may also contain microbial sequences. This exploratory study aimed to investigate the feasibility of prenatal CNV-seq for detecting *Ureaplasma parvum* (*U. parvum*) in amniotic fluids and to evaluate the pregnancy outcomes in *U. parvum*-positive cases.

**Methods:**

This retrospective study enrolled 2419 singleton pregnant women who underwent genetic amniocentesis for fetal CNV-seq testing and completed the follow-up with documented pregnancy outcomes. The CNV-seq data were reanalyzed to extract the read counts of *U. parvum* from each sample’s raw data, and reads per million (RPM) was used to quantify its relative abundance.

**Results:**

The prevalence of asymptomatic intrauterine *U. parvum* positivity in this cohort was 1.4% (33/2419), with read counts ranging 1 to 30423 and RPM from 0.09 to 3580.65 by reanalysis of CNV-seq data. There was a statistically significantly higher risk for early spontaneous preterm labor (<32 gestational weeks; *P*<0.001) and preterm premature rupture of the membranes (*P*<0.001) in women with positive *U. parvum* compared to negative cases. Among *U. parvum* positive cases, six cases (6/33, 18.2%) had relatively higher read counts ranging from 2483 to 30423, with corresponding RPM of 406.45 to 3580.65. Adverse pregnancy outcomes were exclusively observed among women with high reads of *U. parvum* as opposed to those with low reads. Four cases with high *U. parvum* reads in amniotic fluids, not treated with antibiotics, showed a latency period of 6 to 10 weeks from positive detection to the onset of clinical manifestations.

**Conclusions:**

CNV-seq may be a feasible method for detecting intraamniotic *U. parvum* infection. High abundance of asymptomatic *U. parvum* in amniotic fluids are statistically associated with adverse pregnancy outcomes, highlighting its importance in preliminary screening.

## Introduction

Intrauterine infection is associated with a variety of adverse consequences, including spontaneous abortion, preterm premature rupture of the membranes (pPROM), early spontaneous preterm labor (SPB), stillbirth, postpartum hemorrhage, and high rates of neonatal morbidity and mortality (Goldenberg et al., 2000, 2008; Kim et al., 2015; Tita and Andrews, 2010). Microbiological studies have found that vaginal organisms ascending from the cervix to the uterus is the primary route of intrauterine infection, and *Ureaplasma* sp*ecies* (*U.spp*) are the most frequent organisms detected from the amniotic cavity and vagina of women with preterm birth and pPROM (Goldenberg et al., 2008, 2000; Kim et al., 2015; Rittenschober-Böhm et al., 2021, 2019, 2018; Tita and Andrews, 2010). However, the extensive vaginal colonization of *U.spp* among healthy pregnant women without overt clinical manifestations of infection or adverse outcomes has cast doubts on the virulence of *U.spp*, which was even considered as commensal bacteria (Cassell et al., 1993). Consequently, numerous studies based on both animal and human data have been conducted aimed to clarify the pathogenicity of *U.spp*, but yielding mixed and disparate results (Senthamaraikannan et al., 2016; Tantengco et al., 2021; Noda-Nicolau et al., 2022; Bento et al., 2023).

A recent systematic review elucidated several factors contributing to the inconsistent association of *U.spp* with poor pregnancy prognosis, such as microbial biovars, colonization sites, microbial loads, and the methods used for microbial identification (Noda-Nicolau et al., 2022). Notably, two predominate species of *U.spp* in humans are *Ureaplasma parvum (U. parvum)* and *Ureaplasma urealyticum*. Many studies have demonstrated that *U. parvum* has more significant correlation with adverse perinatal outcomes than *Ureaplasma urealyticum* (Kataoka et al., 2006; Uchida et al., 2013; Prince et al., 2016; Payne et al., 2016; Pavlidis et al., 2020). However, some studies found that ascending infection of *U. parvum* alone could not produce massive inflammation leading to preterm labor (Tantengco et al., 2021; Bento et al., 2023; Tripathy et al., 2023). Conversely, direct intra-amniotic inoculation of *U. parvum* in animal models has demonstrated that intrauterine invasion was a major clinical concern than genital colonization (Motomura et al., 2020). Traditional methods like culture and polymerase chain reaction (PCR) can identify *U.spp* colonization, but their sensitivity relies on the microbial loads and they are unable to distinguish between biovars and genotypes. With the application of new molecular microbiological techniques in the field of obstetric infections, such as real-time quantitative PCR (qPCR), 16S ribosomal RNA (rRNA) sequencing, whole genome sequencing (WGS), and metagenomic next-generation sequencing (mNGS), which has become an alternative approach for simultaneous screening of specific and various microbial pathogens with a strain- or species- or genotype-level resolution (Cox et al., 2016; Prince et al., 2016; Payne et al., 2016; Otgonjargal et al., 2018; Rittenschober-Böhm et al., 2019; Elovitz et al., 2019; Chiu and Miller, 2019). However, despite its potential, the high cost and susceptibility to contamination have limited 16S rRNA and mNGS extensive clinical utility for routine screening. Therefore, it is still worth to explore detection methods for high sensitivity, mature technology, and the relative abundance of microbial loads capacity.

As an important diagnostic tool for microdeletion and microduplication syndromes, copy number variation sequencing (CNV‐seq), a next-generation sequencing (NGS) method, has been widely used in prenatal testing for congenital genetic disorders (Liang et al., 2014; Dong et al., 2016; Zhu et al., 2016; Wang et al., 2018b). Given the similar experimental procedure between CNV-seq and mNGS, CNV-seq data may also contain the sequences of microorganisms. Therefore, in this study, we reanalyzed prenatal CNV-seq data in an attempt to detect *U. parvum*, a prevalent pathogenic organism associated with adverse pregnancy outcomes. The primary objective is to evaluate the feasibility and efficacy of detecting *U. parvum* using CNV-seq data. The secondary aim is to explore the nature course and pregnancy outcomes of asymptomatic pregnant women who tested positive for intraamniotic *U. parvum* at the time of genetic amniocentesis.

## Materials and methods

### Study design and populations

This retrospective study enrolled singleton pregnant women who underwent genetic amniocentesis at the Third Affiliated Hospital of Sun Yat-sen University from January 2021 to December 2022. The inclusion criteria for participants were that their collected amniotic fluids were sent for fetal chromosomal karyotyping and CNV-seq, with or without whole exome sequencing under the consent of the pregnant women. The exclusion criteria were as follows: (1) maternal age ≥ 45 years; (2) multiple pregnancies; (3) symptoms of spontaneous miscarriage or preterm labor before amniocentesis, such as vaginal bleeding, abdominal pain, or a shortened cervix; (4) fever or other symptoms of systemic inflammatory response before amniocentesis; (5) infectious diseases, including infections with hepatitis viruses, human immunodeficiency virus, or syphilis; and (6) long-term use of glucocorticoids or immunomodulators. Ethical approval for the study was obtained from the Ethics Committee of our hospital (approval number: II2025-130-01). Prior to amniocentesis, all participants provided written informed consents for the further reanalysis of their experimental and clinical data.

### Clinical data collection

At our hospital, all pregnant women who underwent amniocentesis were prospectively followed up at least three times, including two weeks post-procedure, four weeks post-procedure, and three to six months postpartum. These follow-ups were conducted through a combination of inpatient and outpatient visits, as well as telephone interviews. Follow-ups mainly focused on collecting data on post-procedure complications, pregnancy outcomes, birth defects, and infant growth, all of which were meticulously documented within prenatal diagnosis records. Clinical data of the enrolled pregnant women were obtained from electronic medical records and prenatal diagnosis files. Maternal prenatal information included age, parity, previous obstetrics history, gestational age at the time of amniocentesis, indication for genetic diagnosis, the color of the amniotic fluid, and the results of genetic tests. Pregnancy outcomes included gestational age at delivery, any obstetric complications, and neonatal outcomes.

### CNV sequencing

The experimental steps were as follows: The amniotic fluids were centrifuged at 1500 g for 10 minutes and the resulting cell pellet was used to extract genomic DNA (gDNA) using the QIAamp DNA Mini Kit (QIAGEN, Germany). The gDNA was then fragmented into 200 bp fragments using restriction endonucleases. Libraries were prepared using the CNV-seq Library Preparation Kit (BerryGenomics, China). Library sequencing was performed on the NextSeq CN500 platform (Illumina, USA) using SE36 sequencing mode.

### CNV-seq data reanalysis for *U. parvum*

The CNV-seq data underwent initial processing involving adapter trimming, and reads containing more than three low-quality bases (Q <20) or more than one ambiguous base (N) were removed using an in-house workflow. Subsequently, the remaining clean reads were reanalyzed using the Kraken2 software (version 2.1.2) with Kraken2/Bracken RefSeq PlusPF database (k2_pluspf_20210517) as a reference, established by BenLangmead (https://benlangmead.github.io/aws-indexes/k2). Default parameters were employed for Kraken2 (–minimum-hit-groups 2, –confidence 0.0, -l 31, -k 35). Species of *U. parvum* (taxid:134821) and the corresponding read counts were extracted from the primary dataset of each sample. The depth-based assessment (relative abundance) of *U. parvum* was quantified using normalization method based on the ratio of *U. parvum* read counts to the total reads per million in the CNV-seq dataset, calculated as follows (Qin et al., 2021).


$$\text{Reads per million }\left(\text{RPM}\right)=\frac{\text{Reads count of }U.\;parvum*10^{6}}{\text{Total reads of CNV}-\text{seq data}}$$

### Statistical analysis

The normality of continuous variables was assessed using Shapiro-Wilk test. Continuous data were presented by mean ± standard deviation (SD) or median (interquartile range), with group differences analyzed through Student’s *t* test or Wilcoxon rank-sum test. Categorical data were expressed as numbers (percentage) and analyzed using chi-square test or Fisher’s exact test. Logistic regression analysis was performed to evaluated risk factors associated with SPB. The cumulative distribution of SPB between *U. parvum*-positive and *U. parvum*-negative cases was analyzed using Cox regression. Statistical analysis was performed using SPSS version 27.0 (IBM, USA). A *P* value <0.05 (two-tailed) was considered statistically significant.

## Results

### Study population

**Figure 1:**
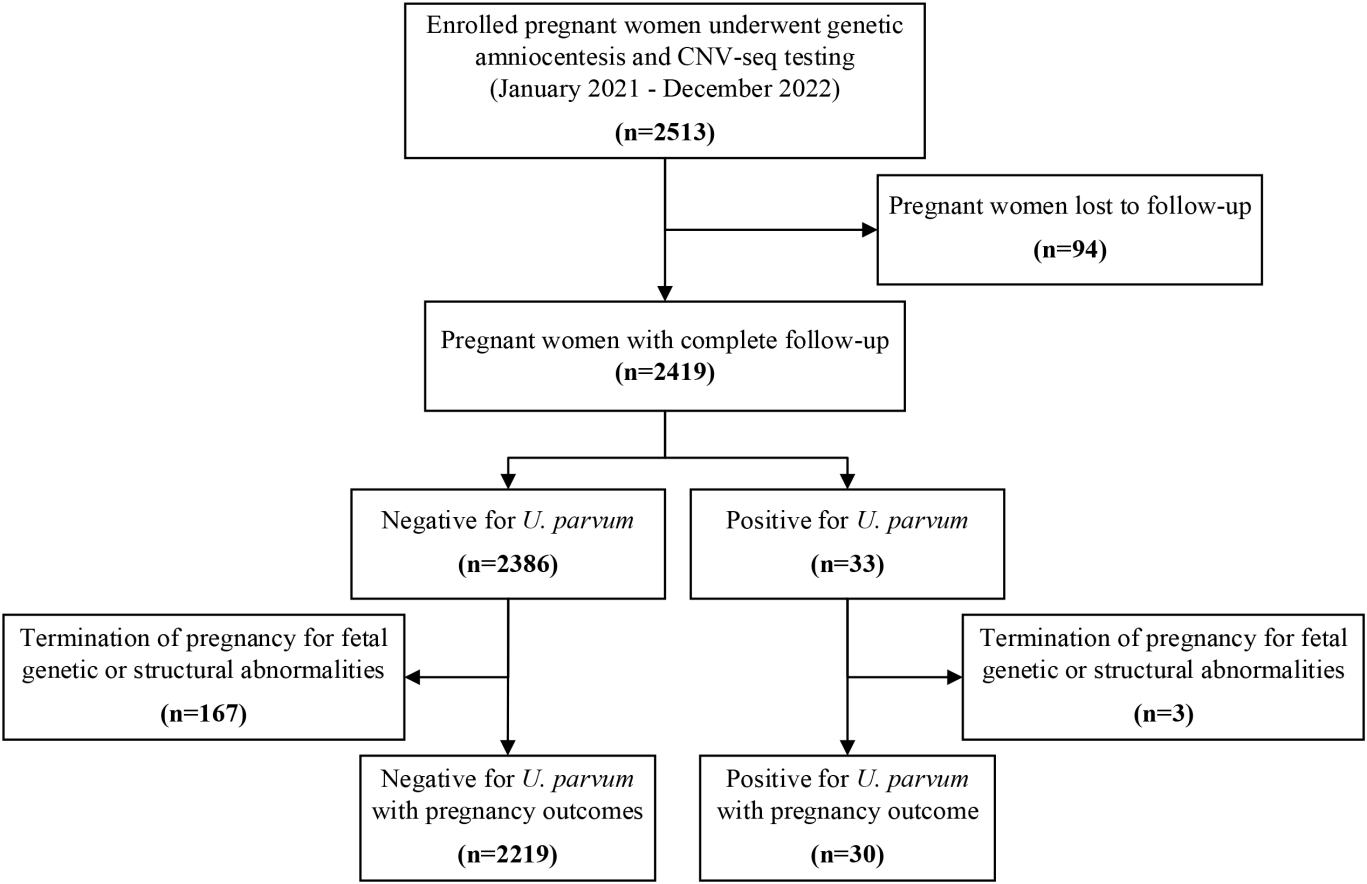
Derivation of the study population.

During the study period, amniotic fluid samples from 2513 singleton pregnancies were analyzed for chromosomal karyotyping and CNV-seq analysis, revealing 82 cases with genetic abnormalities. All cases received follow-up evaluations at two weeks and four weeks post-procedure. While 94 (3.7%) were lost to follow-up after delivery, the remaining 2419 women completed the follow-up and their pregnancy outcomes were documented (**Figure 1**).

### Cases positive for *U. parvum* in amniotic fluids

Upon reanalysis of CNV-seq data from 2419 women, the total sequencing reads per sample ranged from 3.75 million to 20.95 million reads, with an average of 7.08 million reads. Among these, 33 samples (1.4%) showed positive for *U. parvum*, with read counts ranging from 1 to 30423 and RPM from 0.09 to 3580.65. Within this subset, six cases (6/33, 18.2%) displayed a relative higher number of read counts, ranging from 2483 to 30,423, with corresponding RPM of 406.45 to 3580.65. The remaining 27 women showed low reads of *U. parvum*, ranging from 1 to 167, with RPM between 0.09 to 19.74. Meanwhile, among the 33 women with positive *U. parvum*, three underwent mNGS testing, which confirmed that two cases with high reads of *U. parvum* from CNV-seq data were true positive, whereas one case with a single read of *U. parvum* was a false positive.

### Pregnancy outcome of cases testing positive and negative for *U. parvum*

Among the 2386 *U. parvum*-negative cases and 33 *U. parvum*-positive cases with documented outcomes, 167 and three women, respectively, opted for pregnancy termination due to fetal genetic or structural abnormalities. No miscarriage occurred within two-weeks post-procedure in either group. Of the remaining 2219 women tested negative for *U. parvum*, the mean gestational age at delivery was significantly earlier than that of the 30 *U. parvum*-positive cases (*P*<0.001). SPB and pPROM occurred in 107 (107/2219, 4.8%) and 21 (21/2219, 0.9%) women with *U. parvum*-negative, respectively. Conversely, among women with positive *U. parvum*, five (5/30, 16.7%) experienced SPB and four (4/30, 13.3%) suffered pPROM, with rates significantly higher than those in *U. parvum*-negative cohort (*P*<0.001). Of note, in the analysis of gestational age for preterm labor, women testing positive for *U. parvum* were significantly more likely to experience early SPB (<32 weeks gestation) compared to negative cases (*P*<0.001) (**Table 1**).

**Table 1:** Clinical characteristics and outcomes of recruited pregnant women with *U. parvum* positive and *U. parvum* negative.

Parameters	*U. parvum* positive	*U. parvum* negative	*P*
Baseline characteristics	n=33	n=2386	
Maternal age (y), mean ± SD	32.7 ± 5.0	33.66 ± 4.7	0.398
Primipara, n (%)	10 (30.3%)	929 (38.9%)	0.312
GA at amniocentesis (wk), mean ± SD	20.2 ± 3.3	19.9 ± 3.1	0.761
Termination of pregnancy, n (%)	3 (9.1%)	167 (7.0%)	NA
Pregnancy outcomes	n=30	n=2219	
GA at delivery (wk), mean ± SD	36.9 ± 4.8	38.7 ± 1.9	<0.001
pPROM, n (%)	4 (13.3%)	21 (0.9%)	<0.001^a^
SPB, n (%)	5 (16.7%)	107 (4.8%)	<0.001^a^
SPB <32 gestational weeks, n (%)	4 (13.3%)	14 (0.6%)	<0.001^a^
SPB at 32–34 gestational weeks, n (%)	1 (3.3%)	11 (0.5%)	0.149 ^a^

GA, gestational age; pPROM, preterm premature rupture of the membranes; SPB, spontaneous preterm birth; NA, not applicable.

a Fisher’s exact test.

**Figure 2:**
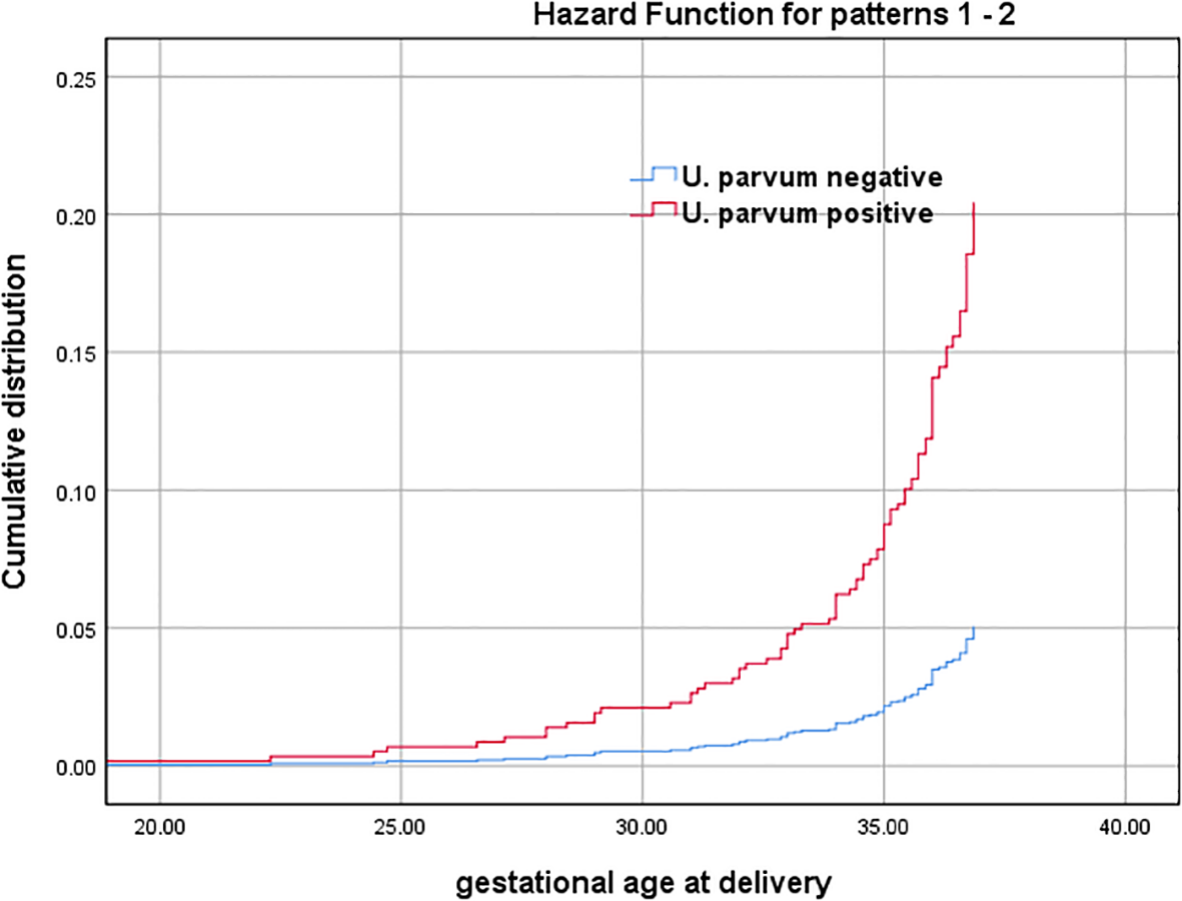
Gestational age at delivery among SPB cases with *U. parvum* positive and *U. parvum* negative (Cumulative distribution curves represent estimated cumulative frequencies of gestational age at birth among pregnant women who suffered SPB with *U. parvum* positive and *U. parvum* negative). Adjusted for maternal age and gestational weeks at amniocentesis.

Multivariate logistic analysis revealed that *U. parvum* positive [adjusted odds ratio (aOR): 3.90, 95% confidence interval (CI): 1.47-10.30, *P*=0.06] was associated with SPB, while maternal age and gestational weeks at amniocentesis showed no significant correlation (*P*>0.05). The subsequence of intraamniotic *U. parvum* infection was further illustrated through the estimated cumulative frequencies of gestational age at delivery among pregnant women who suffered SPB. As depicted in **Figure 2**, the curve for *U. parvum* positive was significantly shifted towards earlier gestational weeks and exhibited higher frequencies compared with *U. parvum* negative. After adjusting for maternal age and gestational weeks at amniocentesis, the presence of *U. parvum* was identified as an independent risk factor for SPB (adjusted hazard ratio (aHR): 4.03, 95% CI:1.64-9.88, *P*=0.002).

When considering the read counts of *U. parvum* from CNV-seq, five out of six (5/6, 83.3%) pregnancies with relatively higher reads resulted in SPB between 24 and 32 gestational weeks, leading to two (2/6, 33.3%) neonatal deaths and one (1/6, 16.7% intrauterine fetal demise at 27 gestational weeks. Histological examination of the placenta was performed in three cases, revealing the presence of chorioamnionitis in all instances. In contrast, among 27 women with low reads of *U. parvum*, three chose termination of pregnancy for fetal genetic or structural abnormalities, while the remaining 24 achieved term deliveries. Detailed clinical data for these 33 cases were presented in **Tables 2** and **3**.

**Table 2:** Detailed clinical data and pregnancy outcomes of 27 cases with low reads of positive *U. parvum*.

Case No.	Assay batch	GA at AC	Indication of AC	Color of amniotic fluid	Genetic testing	Read counts	RPM	Pregnancy outcomes
**1**	B1	20	Fetal left pleural effusion and ascites	Clear	Normal	1	0.16	Amniotic fluid mNGS testing negative. VD at 40 gestational weeks
**2**	B1	19	AMA and high risk of DS	Clear	Normal	11	1.65	VD at 39^+3^ gestational weeks
**3**	B1	19^+6^	AMA and high risk of DS	Clear	Normal	1	0.15	VD at 40^+1^ gestational weeks
**4**	B2	18	AMA and high risk of DS	Clear	Normal	1	0.15	VD at 40 gestational weeks
**5**	B3	18^+2^	High risk of DS	Clear	Normal	2	0.22	CS at 39^+2^ gestational weeks
**6**	B3	20^+1^	AMA	Clear	47, XXX	1	0.11	VD at 39 gestational weeks
**7**	B4	30^+4^	Severe FGR	Clear	Normal	1	0.18	CS at 39^+3^ gestational weeks
**8**	B4	22^+4^	Alpha thalassemia	Clear	Normal	10	1.78	VD at 40 gestational weeks
**9**	B5	21^+5^	Cerebral ventriculomegaly and dilation of bile duct	Clear	Normal	5	0.12	VD at 39^+1^ gestational weeks, received operation due to biliary atresia
**10**	B6	18^+2^	High risk of DS	Clear	Normal	2	0.48	VD at 39^+5^ gestational weeks
**11**	B7	22	Family history of deafness	Clear	Normal	1	0.14	VD at 38^+2^ gestational weeks,
**12**	B8	17	High risk of DS	Clear	Normal	5	0.78	CS at 38^+6^ gestational weeks
**13**	B8	23^+5^	Cerebral ventriculomegaly	Clear	Normal	3	0.29	Termination of pregnancy at 27 gestational weeks due to progression to hydrocephalus
**14**	B8	18^+2^	High risk of DS	Clear	Normal	2	0.26	VD at 39^+4^ gestational weeks
**15**	B8	18	High risk of DS and positive of cfDNA screening	Clear	Trisomy 18	28	3.64	Termination of pregnancy at 20 gestational weeks
**16**	B8	18	Thickened NT	Clear	Normal	6	0.83	VD at 38^+3^ gestational weeks
**17**	B8	18^+3^	AMA and high risk of DS	Clear	Normal	66	5.94	CS at 38^+5^ gestational weeks
**18**	B8	19^+4^	High risk of DS	Clear	Normal	36	5.40	VD at 39 gestational weeks
**19**	B8	18	AMA	Clear	Normal	56	8.00	VD at 38 gestational weeks
**20**	B8	19^+3^	High risk of DS	Clear	Normal	167	19.74	VD at 38^+5^ gestational weeks
**21**	B9	31^+3^	Severe FGR and external genitalia abnormalities	Clear	Normal	1	0.11	Termination of pregnancy at 33^+2^ gestational weeks
**22**	B10	22^+3^	AMA and high risk of DS	Clear	Seq [GRCh37] 2q21.3 (135820000-136120000) ×1, VOUS	6	0.63	VD at 38^+5^ gestational weeks
**23**	B11	19^+3^	Alpha thalassemia	Clear	Normal	1	0.09	CS at 38^+3^ gestational weeks
**24**	B11	17^+6^	High risk of DS	Clear	Seq [GRCh37] 22q12.3 (36340000- 36700000) ×3, VOUS	1	0.13	VD at 39^+4^ gestational weeks
**25**	B12	17^+4^	AMA and high risk of DS	Clear	Normal	6	0.68	VD at 39^+3^ gestational weeks
**26**	B13	19^+6^	AMA and high risk of DS	Clear	Normal	1	0.24	CS at 40 gestational weeks
**27**	B13	19^+5^	AMA and high risk of DS	Clear	Seq [GRCh37] Xp22.31 (6440000-8140000) ×0, Pathogenic	3	0.54	CS at 40 gestational weeks

GA, gestational age; AC, amniocentesis; RPM, reads per million; mNGS, metagenomic next-generation sequencing; AMA, advanced maternal age; DS, Down syndrome; VD, vaginal delivery, CS, Caesarean section; VOUS, variants of uncertain significance; FGR, fetal growth restriction.

**Table 3:** Detailed clinical data and pregnancy outcomes of six cases with high reads of positive *U. parvum*.

Case No.	Assay batch	GA at AC	Indication of AC	Color of amniotic fluid	Genetic testing	Read counts	RPM	Amniotic fluid mNGS or vaginal smear	Pregnancy outcomes
**28**	B1	23^+3^	AMA and high risk of DS	Clear	Normal	30423	2413.37	mNGS with positive *U. parvum* and *Enterococcus faecalis*, vaginal *U. parvum* colonization	Treated with Azithromycin for 5 days until vaginal smear negative; pPROM, SPB at 32^+4^ gestational weeks, VD; Placenta pathology with stage 2 acute chorioamnionitis
**29**	B2	19^+3^	High risk of DS	Clear	Normal	2483	406.45	None	pPROM and SPB at 26^+4^ gestational weeks, VD; Neonatal death
**30**	B4	19^+2^	AMA and high risk of DS	Brown	Normal	14072	3096.26	Vaginal *U. parvum* colonization	Threatened premature labor at 24 gestational weeks; Ultrasound indicated FGR at 25 weeks; intrauterine fetal demise occurred at 27 weeks, VD
**31**	B7	17^+6^	AMA and high risk of DS	Clear	Normal	7337	1013.58	Massive maternal WBC counts, mNGS with positive *U. parvum*, vaginal *U. parvum* colonization	Treated with Azithromycin for 2 weeks until vaginal smear negative; pPROM at 26 gestational weeks; SPB and CS at 28^+3^ weeks; Placenta pathology with stage 2 acute chorioamnionitis
**32**	B8	18^+1^	AMA and high risk of DS	Clear	Normal	15181	1900.16	None	pPROM at 27 gestational weeks; SPB and VD at 27^+3^ weeks
**33**	B13	18^+2^	AMA and high risk of DS	Clear	Normal	20477	3580.65	None	SPB and VD at 24^+5^ weeks; Neonatal death

GA, gestational age; AC, amniocentesis; RPM, reads per million; mNGS, metagenomic next-generation sequencing; AMA, advanced maternal age; DS, Down syndrome; pPROM, preterm premature rupture of the membranes; SPB, spontaneous preterm births; VD, vaginal delivery; CS, Caesarean section.

Regarding the latency period from detection of high reads of *U. Parvum* in amniotic fluids to the onset of overt clinical manifestation, our four cases (case 29, 30, 32 and 33) without antibiotics treatment showed a relative long interval ranging from 6 to 10 weeks. In other two cases (case 28 and 31), Azithromycin was administrated until the vaginal swabs turned negative; however, both cases experienced SPB and pPROM approximately 9 weeks after amniocentesis.

## Discussion

Our reanalysis of prenatal amniotic CNV-seq data showed that the prevalence of *U. parvum* positive in amniotic fluids was 1.4%, with only 0.2% (6/2419) exhibiting high read counts of *U. parvum*. Meanwhile our clinical data indicated a statistically significant association between asymptomatic high reads of *U. Parvum* with subsequent adverse pregnancy outcomes, such as SPB, pPROM, stillbirth, and histological chorioamnionitis. Conversely, the presence of low reads of *U. Parvum* in amniotic fluid did not increase the risk of poor pregnancy outcomes. These findings underscore the potential for detecting and quantifying the relative abundance of *U. Parvum* using prenatal amniotic CNV-seq data without the need for additional tests and associated costs. Additionally, our clinical data showed a relatively long latency period from the detection of subclinical high reads of *U. Parvum* in amniotic fluids to the onset of SPB or pPROM. This observation signifies a nature course of intrauterine *U. Parvum* infection and highlights its chronic progression from infection to subsequent clinical symptoms.

It is well-established that intrauterine infection is considered the main cause of preterm birth and pPROM (Goldenberg et al., 2000; Romero et al., 2007; Goldenberg et al., 2008), with the majority of cases beginning as subclinical (Romero et al., 2014). The most common organism isolated from the amniotic cavity is *U.* spp, with the reported positive rate ranging from 11.4 to 18.4% among asymptomatic women at mid-trimester (Gerber et al., 2003; Perni et al., 2004), and from 22% to 43.9% in cases complicated with preterm birth or pPROM from early studies (Witt et al., 2005; Kirchner et al., 2007; Kasper et al., 2010). Moreover, both direct and indirect causal relationships between *U.* spp and preterm birth has been confirmed by numerous human pregnancies and animal models (Gerber et al., 2003; Perni et al., 2004; Witt et al., 2005; Kasper et al., 2010; Viscardi, 2010; Uchida et al., 2013; Yoneda et al., 2016; Motomura et al., 2020). Consistent with these studies, our analysis of prenatal amniotic CNV-seq data revealed that asymptomatic positive of *U. Parvum*, particularly in high reads of case series, significantly linked to a spectrum of adverse pregnancy outcome including SPB, pPROM, stillbirth, and histological chorioamnionitis. However, a multicenter randomized trial in France tried to assess the effectiveness of antibiotics treatment in asymptomatic second-trimester women with *Mycoplasma* or *U.* spp positivity, and found a relatively low rate (3.0%) of amniotic fluid colonization which was not associated with any adverse pregnancy or neonatal outcomes (Kayem et al., 2018). Nevertheless, two recent systematic review and meta-analysis investigating *U.* spp’s pathogenic potential for early deliveries, but yielded unexpectedly inconsistent results (Noda-Nicolau et al., 2022; Jonduo et al., 2022). An important reason for this may be the heterogeneous bacterial detection methods and differences in sample types among the eligible studies.

Prior to 2010, conventional culture methods were predominantly used for detection of *U.* spp in the early reported studies (Gerber et al., 2003; Perni et al., 2004; Witt et al., 2005; Kirchner et al., 2007). However, a research team from South Korea found that more than one third of amniotic microbial invasion with *U.* spp was missed by culture method when compared with PCR technique, and these cases with negative culture but positive through PCR were also associated with high risk of adverse outcomes (Yoon et al., 2000, 2003). Afterwards, many studies had revealed that modern molecular biological methodologies not only can discriminate different biovars and genotypes of *U.spp*, but also can detect bacterial loads compared to traditional culture-based methods (Kim et al., 2003; Kasper et al., 2010; Xiao et al., 2011; Yoneda et al., 2016; Prince et al., 2016; Payne et al., 2016; Otgonjargal et al., 2018; Rittenschober-Böhm et al., 2019; Stranik et al., 2022). Meanwhile, those findings also showed that intrauterine infection with *U. parvum* was the predominant biovar and significantly associated with adverse perinatal outcomes (Cox et al., 2016; Prince et al., 2016; Otgonjargal et al., 2018; Motomura et al., 2020). Even with a very low positive rate (1.4%) of *U. Parvum* from amniotic fluid, the present prenatal data found that all six asymptomatic cases with high reads of this microorganism during second trimester ultimately experienced preterm delivery at very early gestational age between 24 to 32 weeks. Therefore, our data provides additional evidence that the abundant invasion of *U. Parvum* in the amniotic cavity possesses intensive pathogenic potential for clinical adverse consequences associated with intrauterine infection.

Regarding quantification of bacterial loads within the uterine cavity, several microbiological methods have been developed, including qPCR, 16S rRNA sequencing, and mNGS (Jacobsson et al., 2009; Kasper et al., 2010; DiGiulio et al., 2010; Kacerovsky et al., 2012; Aagaard et al., 2014; Sweeney et al., 2016). qPCR is particularly noted for its rapid and sensitive quantification of specific microbial DNA in clinical samples, also provides valuable insights into microbial burden levels. However, the limitation of qPCR is reliance on predetermined target sequences, potentially missing unrecorded pathogens and lacking a detailed microbial profile. The modern techniques of 16S rRNA sequencing and mNGS, on the other hand, offer a comprehensive assessment of the microbial composition and diversity within a specific compartment, also providing the relative abundance of different microorganisms simultaneously by counting sequenced reads in the sample (Kembel et al., 2012; Bertelli and Greub, 2013; Chiu and Miller, 2019). This capability is essential for assessing the severity of intrauterine infections and predicting their related adverse pregnancy outcomes (Jacobsson et al., 2009; Kasper et al., 2010; Kacerovsky et al., 2011, 2012). Nonetheless, 16S rRNA lacks detailed quantitative data at the species level, while mNGS requires significant computational resources and may be cost-prohibitive for routine clinical use (Deurenberg et al., 2017; Johnson et al., 2019). Similar to the process of mNGS, CNV-seq technique may also detect microbial sequences. Our data confirmed this conception and also demonstrated a close link between the high burden of *U. Parvum* in the amniotic cavity and adverse outcomes. Conversely, cases with low levels of *U. Parvum* consistently resulted in uneventful term deliveries. These contrasting results were mainly attributed to different microbial loads in the amniotic cavity. It is well understood that the degree of microbial load directly correlates with the intensity of inflammatory response, and higher microbial burdens tend to trigger a more robust immune response, often leading to significant complications (Jacobsson et al., 2009; Kasper et al., 2010).

Another potential explanation for our observations of low concentrations of intrauterine *U. Parvum* presence could be false-positive results. Given that modern molecular technologies are highly sensitive and capable of detecting very small amounts of nucleic acid, the testing process is relatively prone to contamination and makes the results susceptible to false-positive (Zinter et al., 2019; Cantalupo and Pipas, 2019; Du et al., 2022). We noted that cases 1-3, 4, 7-8, 11, 12-20, and 26–27 were processed for CNV-seq testing in the same assay batch as one of six samples with high microbial loads, raising suspicious of cross-contaminated from the severely infected samples due to index swapping (Costello et al., 2018). For the other nine cases with low read counts, ranging from 1 to 6, we considered the possibility of non-specific read mapping. However, verifying false-positive through bioinformatics analysis to establish thresholds for sequence reads and RPM is both technique-intensive prior to testing and time-consuming afterwards (Chiu and Miller, 2019). Unfortunately, retrospective data is not feasible for this purpose. Another possible explanation could be a mild intrauterine *U. parvum* infection that was effectively cleared by the host immune system without causing any clinical symptoms and subsequent complications. This suggests that intrauterine presence of *U. parvum* is not necessarily indicative of adverse pregnancy outcomes (Rodríguez et al., 2011; Kayem et al., 2018). Therefore, interpreting these low read counts of intrauterine microbial presence demands considerable cautions.

Given the chronicity of intrauterine infections (Goldenberg et al., 2000, 2008), early detection of microbial presence in the amniotic cavity is theoretically crucial for timely and effective antibiotics treatment. Our four clinical cases (case 29, 30, 32 and 33) without antibiotics treatment exhibited latency periods ranging from 6 to 10 weeks from the detection of subclinical high reads of *U. Parvum* in amniotic fluids to overt clinical manifestation. This finding further highlights the chronic nature of intrauterine infection, from the detection of *U. Parvum* positive in mid-trimester to the emergence of overt clinical symptoms, thereby providing sufficient time for clinical antibiotic intervention. However, despite the timely administration of antibiotics until the vaginal swabs turned negative, SPB and pPROM still occurred in other two cases (case 28 and 31). These findings suggested that antibiotics treatment may not completely eradicate severe amniotic infections, particularly with drugs like Azithromycin, which have limited ability to cross the placental barrier (Heikkinen et al., 2000; Witt et al., 2003).

As an untargeted microbial pathogen screening tool, mNGS boasts significant advantages over traditional microbial culture and has been increasingly applied in clinic for patients with severe or rare infections (Gu et al., 2019; Sun et al., 2022). It predominantly uses host-cell-free samples such as plasma, cerebrospinal fluid, or bronchoalveolar lavage fluid, and often involves the removal of host DNA to enrich microbial genomes, thereby enhancing its sensitivity (Blauwkamp et al., 2019; Miller et al., 2019; Huang et al., 2020). For optimal sensitivity, mNGS data should consist of no fewer than 10 million sequencing reads, with some studies even up to 36 million reads (Li et al., 2021). In contrast, CNV-seq, designed for detecting CNVs in the human genome, does not intentionally enrich microbial genomes and generally requires only a minimum of 3 million sequencing reads (Zhu et al., 2016; Wang et al., 2018b; Hayes et al., 2013; Wang et al., 2018a; Meng and Jiang, 2022). As such, despite a similar testing process to mNGS, the suitability of CNV-seq data for analyzing microbial pathogens remains uncertain. Notably, in this study cohort, we identified six cases from 2419 prenatal samples with high read counts of intrauterine *U. parvum*, whose clinical manifestations and outcomes were consistent with previous reported intrauterine *U. parvum* infection (Gray et al., 1992; Gerber et al., 2003; Perni et al., 2004; Witt et al., 2005; Kirchner et al., 2007; Kasper et al., 2010). Due to the low-depth sequencing of CNV-seq, the definitive connection between *U. parvum* colonization and adverse outcomes should be interpreted with cautions, and a larger sample size and additional research are needed to further validate the performance of CNV-seq in detecting *U. parvum* and other microorganisms.

The primary strength of our study lies in our innovative reanalysis of prenatal CNV-seq data to detect *U. parvum* intrauterine infection, providing a novel method for identifying early infection beyond prenatal genetic diagnosis. To our knowledge, this study represents the first attempt to evaluate the feasibility and efficacy of using CNV-seq data for detecting *U. parvum* in amniotic fluids. Give these results, this approach could theoretically be extended as an initial screening tool for other latent intrauterine microbial pathogens after amniocentesis, particularly in cases presenting with fetal growth restriction, ventriculomegaly, and fetal hydrops. In such scenarios, reanalyzing existing CNV-seq data might offer valuable microbial insights without the need for universal mNGS screening, thereby reducing the medical burden on these pregnant women.

However, our study had several limitations that should be acknowledged. First, CNV-seq testing does not involve microbial enrichment, and the number of sequencing reads is approximately one-third that of mNGS, which would inevitably reduce its sensitivity in detecting microbial infections. Second, due to the absence of orthogonal clinical testing and external no-template control samples, current retrospective data could not conclusively determine whether the detection of low abundance *U. parvum* represented a true or false positive, nor confirm the thresholds for relatively high and low read counts. Third, given the limited numbers of cases with positive *U. parvum* detection and wide variations in read counts and corresponding RPM values, it is not feasible to perform correlation analysis or determine predictive values for adverse pregnancy outcomes. Lastly, the absence of standard methods such as qPCR, 16S rRNA, and mNGS as controls hinders our ability to accurately determine the precise intrauterine infection rate of *U. parvum* in the study population.

## Conclusion

It is feasible to detect and quantify the relative abundance of *U. parvum* in amniotic fluids using CNV-seq data. Asymptomatic high intrauterine *U. parvum* loads are statistically linked to adverse pregnancy outcomes, particularly an increased risk of early SPB. Additionally, CNV-seq has potential as a preliminary screening approach for other microbes, particularly beneficial for pregnant women who are at high risk of intrauterine infections and require prenatal genetic amniocentesis. Important directions for future research include the following: (1) Optimization of *U. parvum* detection methods through incorporation of independent validation techniques, such as qPCR, culture, or targeted metagenomic sequencing, to confirm the presence of *U. parvum* and assess potential false positives or negatives in CNV-seq data. (2) Enhancement of abundance quantification accuracy by utilizing tools like Bracken2 or other abundance refinement methods in conjunction with Kraken2. (3) Implementation large-scale prospective longitudinal studies addressing additional confounding factors, including maternal health conditions, co-infections, antibiotic use, gestational age, and inflammatory markers, to investigate how initial *U. parvum* abundance and its temporal changes influence the progression and severity of adverse pregnancy outcomes.

## Data Availability

The CNV-seq data from all cases have been deposited in the National Genomics Data Center (NGDC), with the submission accession number HRA011914 (bioProject accession: PRJCA041124; https://bigd.big.ac.cn/gsa-human/browse/HRA011914). Datasheets generated by Kraken2 are available from the corresponding author upon reasonable request.
